# Mitral Valve Repair of Atrial Functional Mitral Regurgitation in Heart Failure with Preserved Ejection Fraction

**DOI:** 10.3390/jcm9113432

**Published:** 2020-10-26

**Authors:** Zsuzsanna Balogh, Takuya Mizukami, Jozef Bartunek, Carlos Collet, Monika Beles, Marzia Albano, Asim Katbeh, Filip Casselman, Marc Vanderheyden, Guy Van Camp, Frank Van Praet, Martin Penicka

**Affiliations:** 1 Cardiovascular Center, OLV Clinic, 9300 Aalst, Belgium; zsuzsi.liliom@gmail.com (Z.B.); mizukami.tky@gmail.com (T.M.); jozef.bartunek@telenet.be (J.B.); carloscollet@gmail.com (C.C.); monika.beles@olvz-aalst.be (M.B.); marz.albano@gmail.com (M.A.); asimkatbeh@yahoo.com (A.K.); marc.vanderheyden@olvz-aalst.be (M.V.); guy.van.camp@olvz-aalst.be (G.V.C.); 2 Department of Cardiovascular and Thoracic Surgery, OLV Clinic, 9300 Aalst, Belgium; filip.casselman@olvz-aalst.be (F.C.); frank.van.praet@olvz-aalst.be (F.V.P.)

**Keywords:** mitral regurgitation, mitral valve repair, heart failure, outcome

## Abstract

Our objective was to describe the long-term effects of endoscopic mitral valve (MV) repair on outcome in patients with heart failure with preserved ejection fraction (HFpEF) and atrial functional mitral regurgitation (AFMR). In patients with HFpEF, even mild AFMR has been associated with poor outcome. The study population consisted of consecutive patients with HFpEF (left ventricular ejection fraction (LVEF) ≥ 50%, H_2_FPEF score ≥ 5) and AFMR, who underwent isolated, minimally invasive endoscopic MV repair (MVRepair group) (*n* = 131) or remained on standard of care (StanCare group) (*n* = 139). Patients with coronary artery disease or organic mitral regurgitation (MR) were excluded. Patients were matched using inverse probability of treatment weighting. Endpoints were all-cause mortality and a composite of all-cause mortality and HFpEF readmissions. The median follow-up was 5.03 years (interquartile range (IQR) 2.6–7.9 years). In the MVRepair group, the perioperative, 30-day, 1-year, and 5-year mortality were 0, 1%, 1%, and 12%, respectively. Additionally, 13 (10%) patients were readmitted for worsening HFpEF, while 2 (1%) individuals underwent redo MV surgery for recurrent MR. MVRepair compared with StanCare showed 21–29% (Standard Error (SE) 6–8%) and 19–26% (SE 6–8%) absolute risk reduction of all-cause mortality and HFpEF readmissions, respectively (all *p* < 0.05). MVRepair emerged as the strongest independent predictor of all-cause mortality (Hazard Ratio (HR) 0.16, 95% (Confidence Interval (CI) 0.07–0.34, *p* < 0.001) and HFpEF readmissions (HR 0.21, 95% CI 0.09–0.51, *p* < 0.001). At 5-year follow-up, in the MVRepair group, a total of 88% were alive and 80% were alive without readmission for HFpEF. We can conclude that endoscopic MV repair is associated with low perioperative mortality as well as high long-term efficacy, and appears to improve clinical outcome in patients with AFMR and HFpEF.

## 1. Introduction

Atrial functional mitral regurgitation (AFMR) is highly prevalent and associated with worse outcome in heart failure patients with preserved ejection fraction (HFpEF) and atrial fibrillation (AF) [1,2,3,4,5]. The optimal treatment strategy for AFMR is not known. Two small studies have reported good short-term effects of undersized mitral valve (MV) annuloplasty using open-chest sternotomy [6,7]. However, the long-term effects of MV intervention on survival are not available. Moreover, patients with HFpEF and AFMR are usually elderly with frequent comorbidities, for whom a minimally invasive technique may provide a distinct safety advantage over sternotomy [1,2].

Video-assisted endoscopic MV repair is a minimally invasive surgical approach using the right chest while avoiding sternotomy [8]. The endoscopic technique shows comparable results to open-chest MV surgery, but with less postoperative morbidity [8]. In ventricular FMR, isolated endoscopic MV annuloplasty conferred an independent long-term survival benefit in patients with heart failure with reduced ejection fraction compared with the guideline-directed therapy [9]. Therefore, the aim of the present study was to investigate the long-term outcomes of isolated endoscopic MV repair in patients with HFpEF and AFMR.

## 2. Methods

### 2.1. Study Design

A retrospective, single-center study.

### 2.2. Study Population

**Figure 1:**
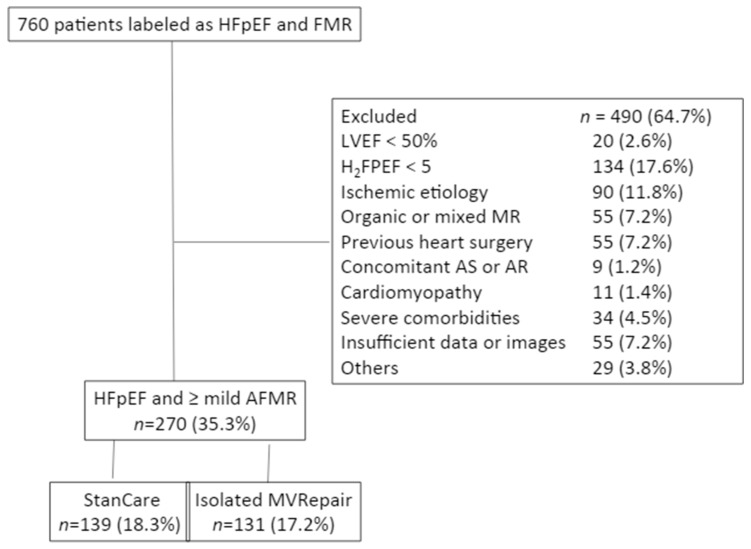
Identification of the study group. All patients labeled as heart failure with preserved ejection fraction (HFpEF) with at least mild functional mitral regurgitation (FMR) have been considered for the study. Abbreviations: AFMR: atrial functional mitral regurgitation; AR: aortic regurgitation; AS; aortic stenosis; LVEF: left ventricular ejection fraction; MR: mitral regurgitation; MVRepair: mitral valve repair group; StanCare: standard of care group.

The study population consisted of consecutive patients with HFpEF and AFMR admitted to our department center due to de novo heart failure (standard of care group, StanCare, *n* = 354) or those undergoing isolated, minimally invasive endoscopic MV repair due to significant persistent AFMR (MVRepair group) between 2003 and 2017 (*n* = 406). The diagnosis of HFpEF was based on clinical presentation and the H_2_FPEF score [10]. AFMR was defined by a structurally normal valve with normal leaflet motion. Mitral regurgitation was graded semi-quantitatively as mild (1/4), moderate (2/4), moderate-to-severe (3/4), or severe (4/4). All individual files and available images were reviewed by an experienced echocardiographer (Z.B.), who was blinded to clinical outcome. To be eligible for the study, patients had to fulfill the following criteria: (1) left ventricular ejection fraction (LVEF) ≥ 50%; (2) H_2_FPEF score ≥ 5 suggesting >80% probability of HFpEF; and (3) non-ischemic etiology of HFpEF. Patients were excluded if they had reduced LV ejection fraction (<50%), history of myocardial infarction, previous heart surgery including myocardial revascularization, suspected ischemic heart disease, need for concomitant myocardial revascularization or aortic valve surgery, congenital heart disease, hypertrophic or restrictive cardiomyopathy, metastatic cancer or other significant comorbidities likely to limit survival, or and unavailable or poor-quality echocardiographic images (Figure 1). The MVRepair group consisted of 131 patients (72 ± 7 years, 21% males) undergoing endoscopic MV repair using undersized semi-rigid annuloplasty rings on an elective basis. Patients with annulus sizes > 32 mm were excluded. No patients underwent myocardial revascularization. Concomitant tricuspid valve annuloplasty or maze procedure was not an exclusion criterion. Functional tricuspid valve regurgitation was treated with tricuspid valve annuloplasty using a ring size of 30 or 32 in males and a ring size of 28 or 30 in females. All patients received left-sided maze set including an endocardial box lesion around the four pulmonary veins with extension to the left atrial appendage and mitral valve annulus. Some patients underwent right-sided Cox maze lesions which were also performed with cryothermia. Right atrial incision was made from superior to inferior vena cava (intercaval line) and extended slightly on the lateral atrial wall. The lateral atrial wall line was then prolonged with a cryo probe towards the lateral border of the tricuspid valve annulus. An extra cryothermia line was performed inside the right atrium from the medial border of the tricuspid valve annulus towards the tip of the right atrial appendage.

The StanCare group consisted of 139 patients (78 ± 9 years, 28% males) hospitalized for de novo HFpEF who showed at least mild AFMR at fully compensated state at discharge. The study protocol was performed in accordance with the Ethics Committee of our institution. The need for consent to participate in this research study was waived in view of its observational and anonymous nature. Demographic data, medical history, laboratory results, imaging findings, periprocedural, and follow-up data were collected for the analysis. In all patients, survival status at the end of follow-up was verified using the national population registry. The cause of each readmission was reviewed in patient records and, if needed, validated with the patient or family doctor.

### 2.3. Statistical Analysis

Data are expressed as mean ± SD for continuous variables and as percentages for categorical variables. The unpaired or paired Student’s *t*-test and Pearson’s correlation coefficient were used as appropriate. Fisher’s exact test was used to compare categorical variables in 2 × 2 contingency tables. Cumulative survival curves were derived according to the Kaplan–Meier method, and differences between curves were analyzed by the log-rank test. Several analyses were performed to estimate the effect of MVRepair on all-cause mortality, HFpEF readmissions, and its composite. (1) Two propensity scores were performed, one based on the perioperative risk (EuroSCORE II, age, NYHA class) and a second based on relevant clinical characteristics (age, gender, NYHA class, body mass index, hypertension, diabetes mellitus, LV end-diastolic diameter, tricuspid regurgitation grade, and pulmonary artery pressure). Scores were used for appropriate local optimal 1:1 caliber matching without replacement using 0.20 caliper width of the logit of the standard deviation of the calculated score to balance the baseline covariates. (2) Kaplan–Meier-derived survival analysis was conducted using inverse probability weighting. (3) Cox regression modelling was done, adjusted for propensity score. Care was taken to avoid overfitting. Considering competing risk events between mortality and rehospitalization, cumulative incidence functions (CIFs) were calculated using Fine and Gray regression for the equality of CIFs in treated groups. For all tests, values of *p* < 0.05 were considered significant. For statistical analysis, R 3.5.0 was used.

## 3. Results

### 3.1. Baseline Characteristics

Table 1 shows unadjusted baseline clinical and echocardiographic characteristics in both groups. Per eligibility criteria, all patients had a non-dilated left ventricle (LV) with preserved LV ejection fraction. The average H_2_FPEF score was >6 in both groups. The majority of patients had a history of AF. The StanCare individuals were significantly older and had higher prevalence of comorbidities compared with the MVRepair group (*p* < 0.01). Despite being low in both groups, the EuroSCORE II was significantly higher in the StanCare versus the MVRepair group (*p* < 0.001). The majority of patients (71%) undergoing MV repair had NYHA class III/IV symptoms. We observed significantly larger left atrial diameter, greater degree of AFMR, and tricuspid regurgitation in patients undergoing MVRepair compared with StanCare (all *p* < 0.001).

### 3.2. Periprocedural and 30-Day Outcome in the MVRepair Group

The MV repair was successful in all patients, and no individuals died during surgery (intraoperative mortality = 0). The median annulus size was 28 mm (range 26–32 mm). Concomitant tricuspid valve annuloplasty and maze was performed in 68 (49%) and 97 patients (70%), respectively. The average cardiopulmonary bypass and myocardial ischemic time was 136 ± 37 min and 93 ± 30 min, respectively. One patient died during index hospitalization from multiorgan failure (hospital and 30-day mortality = 1%). Major surgical complications were observed in a total of 13 (9%) individuals, of whom 9 patients (6%) showed total AV-block requiring implantation of a permanent pacemaker. One patient (1%) had multiorgan failure, 2 (2%) had stroke, and 1 (1%) had sepsis.

### 3.3. Long-Term Outcome

**Figure 2:**
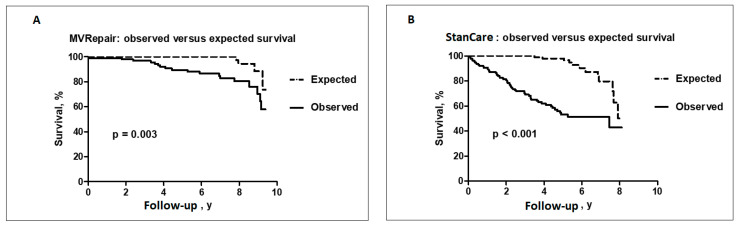
Observed versus expected survival in the MVRepair (**A**) and StanCare groups (**B**).

Follow-up for all-cause mortality and HFpEF readmission was obtained in 100% of patients. During median follow-up of 5.03 years (IQR 2.6–7.9 years) a total of 86 (32%) patients died from any cause and 61 (23%) were readmitted for worsening HFpEF. In the MVRepair group, two patients (1.4%) had redo MV surgery for recurrent MR. In the StanCare group, no patients had MV intervention during the follow-up. Both groups showed significantly shorter time to observed versus expected all-cause mortality in age and sex-matched general population (Figure 2A,B). However, in the MVRepair group, significant separation of the survival curves was observed only after 3 years following MV intervention. In contrast, in the StanCare group, the separation of the survival curves started immediately after index admission. Patients in the StanCare group had significantly higher 1-year (9% vs. 1%, *p* = 0.002) and 5-year (40% vs. 12%, *p* < 0.001) mortality from any cause and 1-year (17% vs. 4%, *p* = 0.006) and 5-year (34% vs. 10%, *p* < 0.002) readmissions for worsening HFpEF, respectively. The total number of HFpEF readmissions was significantly higher in the StanCare versus the MVRepair group (56% vs. 13%, *p* < 0.001).

**Figure 3:**
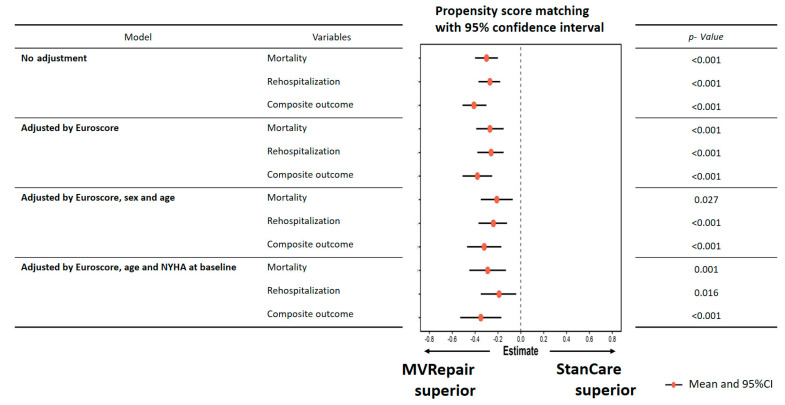
Estimating effect of MVRepair using propensity score matching at 5-year follow-up.

**Figure 4:**
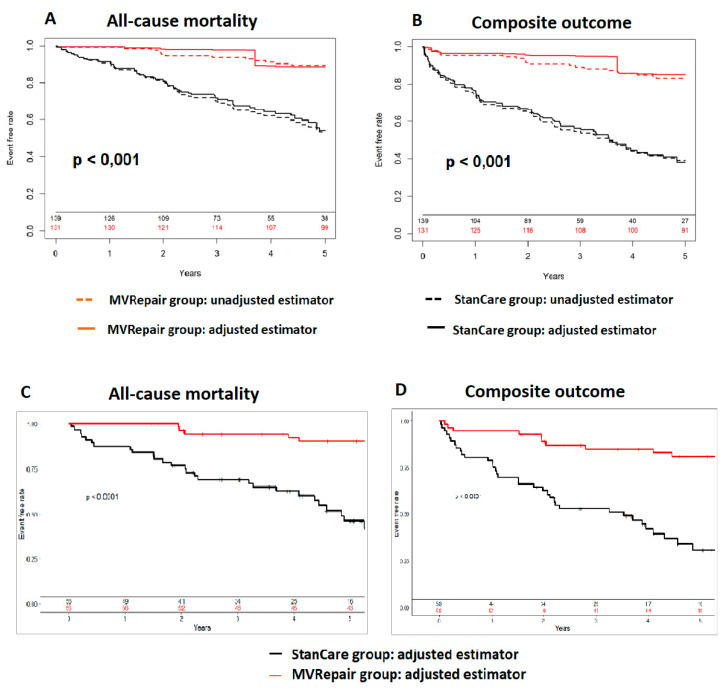
Survival analysis using inverse probability weighting. All-cause mortality (**A**) and its composite with HFpEF readmissions (**B**) after propensity matching for baseline perioperative risk (EuroSCORE II, age, and HYHA class) at 5 years. Unadjusted (dashed lines) and adjusted (solid lines) in the MVRepair (red color) versus the StanCare (black color) groups. Propensity matching resulted in 52 matched pairs. All-cause mortality (**C**) and its composite with HFpEF readmissions (**D**) following propensity matching for relevant clinical characteristics (age, gender, NYHA class, body mass index, hypertension, diabetes mellitus, LV end-diastolic diameter, tricuspid regurgitation grade, and pulmonary artery pressure). The MVRepair group is shown in red, while the StanCare group is shown in black.

**Figure 5:**
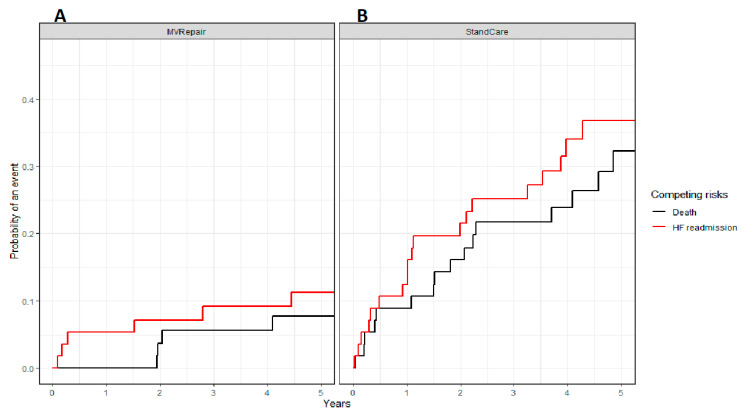
Estimated cumulative incidence curves for all-cause mortality (black) and HFpEF readmissions (red) as competing events for MVRepair (**A**) and StanCare (**B**) groups in population with propensity score matching.

This relationship was significant both in milder and more severe AFMR. The all-cause mortality was significantly lower in the MVRepair versus the StanCare group, both in milder MR (21% vs. 41%) and in more severe MR (24% vs. 50%) (*p* < 0.05). The same trend was observed for HF readmissions, both in milder MR (8% vs. 35%) and more severe MR (11% vs. 33%) (*p* < 0.05). In propensity score analyses, MVRepair compared with StanCare showed significant absolute risk reduction of all-cause mortality (21–29%), HFpEF readmissions (19–26%), and their composite (32–38%) (all *p* < 0.05) (Figure 3 and Figure 4). In Fine and Gray regression analysis, considering competing risk between mortality and HFpEF readmissions, the MVRepair group compared to StanCare group maintained its significantly lower mortality (*p* = 0.010) and HFpEF readmission (*p* = 0.002) (Figure 5).

### 3.4. Predictors of Outcome

MV repair was the strongest independent predictor of all-cause mortality, HFpEF readmissions, and their composite (all *p* < 0.001). The other independent predictors were age and body mass index for all-cause mortality, tricuspid regurgitation grade, and diabetes mellitus for HFpEF readmissions, and age for their composite (Table 2).

## 4. Discussion

The outcomes and durability of isolated mitral valve repair in heart failure patients with atrial functional mitral regurgitation are unknown. The present study indicates that in these patients, isolated endoscopic MV repair of AFMR yields: (1) low perioperative and 30-day mortality, (2) high long-term efficacy with low rate of recurrent AFMR, and (3) reduction of excessive mortality and HFpEF readmissions during follow-up.

### 4.1. Prevalence and Outcome of AFMR

AFMR is a distinct type of mitral regurgitation characterized by structurally normal MV with normal leaflet motion [1,3]. The primary underlying mechanism involves mitral annulus dilatation, either alone, or in some cases combined with an insufficient compensatory leaflet growth or impaired atrial and annular dynamics [1,3,11,12,13,14,15]. AFMR shows high prevalence in patients with HFpEF and AF [1,2,5]. This is related to synergistic hemodynamic and structural changes featuring HFpEF condition. HFpEF leads to increased left atrial pressure and aggravates left atrial remodeling with electrical instability leading to AF. This in turn causes mitral annulus dilatation leading to AFMR associated with worsened symptoms and outcome [1,2,5]. As the prevalence of both HFpEF and AF is steadily increasing, AFMR may become the most frequent type of MR in the near future [1,16,17]. In the ATTEND (acut decompensated heart failure syndromes) registry including 1825 patients with HFpEF, a total of 71% of subjects had at least mild AFMR before discharge following admission for acute decompensation [5]. The presence of even mild ischemic AFMR at discharge has been independently associated with higher occurrence of the composite of all-cause death and HFpEF readmissions [4,5]. In the community patients with isolated moderate-to-severe MR, AFMR accounted for 27% of cases [2]. Note that, despite having relatively small effective regurgitant orifice area (0.2 ± 0.08 cm^2^), AFMR has been associated with excess 5-year mortality (50%) compared with expected mortality in the general population (HR 1.88) [2]. This suggests that even small amounts of regurgitant volume may have prognostic impact in non-dilated and non-compliant LV, alluding to the recently proposed concept of disproportionate FMR [18]. In the present study, we observed even higher prevalence of moderate to severe AFMR (37%), possibly related to the tertiary character of the center and the inclusion of patients with high H_2_FPEF score, AF, and at least mild AFMR [2,5]. In the StanCare group, we observed slightly better 5-year survival than in the Mayo clinic cohort, which may be explained by exclusion of ischemic AFMR from the present study [2].

### 4.2. Management of AFMR

Despite being associated with poor outcome, the optimal therapeutic strategy in AFMR has not been determined [19]. Diuretics and early restoration of sinus rhythm may reduce left atrial pressure and mitral annular area, and hence the severity of AFMR. However, the frequently associated renal impairment and persistence of AF makes this approach challenging for a large proportion of HFpEF patients [20]. Undersized MV annuloplasty targets the major underlying mechanism of AFMR (i.e., annulus dilatation). Two smaller studies totaling 57 patients with permanent AF and preserved LV ejection fraction reported good mid-term effects of MV annuloplasty using the open chest technique [6,7,21]. During median follow-up of 932 days, the rates of composite of freedom from cardiac death and HFpEF readmissions was 64% [21]. In our study, we report the long-term outcome in the largest cohort of HFpEF undergoing MVRepair for AFMR so far. At 5-year follow-up, a total of 88% of patients were alive and 80% were alive without HFpEF readmission, which is a significantly better outcome than previously reported [21]. Furthermore, in our study MVRepair maintained its association with improved survival compared with standard of care even after propensity matching. The more favorable outcome in the current study may be related to its minimally invasive approach, which may present an important safety advantage in frail patients.

### 4.3. Limitations

The present study was retrospective, with all inherent limitations. However, all files and images were carefully examined by an experienced echocardiographer, and patients with insufficient data or poor image quality were excluded. Moreover, in all patients, follow-up for mortality and HFpEF readmissions was obtained.

The information on medical therapy was not systematically collected. At this moment, no medication has been shown to improve survival in HFpEF or in AFMR. Therefore, this information may not limit the extensibility of results.

In the present study, all patients received undersized MV annuloplasty. In rare cases this technique may be associated with mitral valve stenosis. However, in the present study, we did not observe increased MV gradient in any patient. In elderly patients we might consider MV replacement from the start rather than risk an unsuccessful repair. This will depend largely on the patient profile and is based on the surgeon’s operative experience. The MV replacement would not shorten the clamp and ischemic times compared to a valve repair procedure. However, it would certainly be shorter than first performing a valve repair that proves to be inadequate and then needs to be followed by a valve replacement.

## 5. Conclusions

The results of the present study suggest that isolated MV repair is safe and provides long-term benefits over standard of care. In particular, it appears to reduce excess mortality associated with HFpEF and AFMR and reduce the number of unplanned HFpEF admissions. These results should be validated in prospectively designed clinical trials.

## Figures and Tables

**Table 1:** Unadjusted baseline clinical and echocardiography characteristics.

Variables	MVRepair *n* = 131	StanCare *n* = 139	*p*
Age, y	71.99 (7.26)	77.71 (8.92)	<0.001
Gender, *n* (%)	28 (21.4)	39 (28.1)	0.259
Hypertension, *n* (%)	75 (60.5)	90 (64.7)	0.558
Diabetes mellitus, *n* (%)	1 (0.8)	13 (9.5)	0.003
COPD, *n* (%)	13 (10.0)	22 (15.8)	0.216
MDRD < 50 mL /min/1.73 m^2^, *n* (%)	29 (22)	46 (33)	0.057
Stroke, *n* (%)	3 (2.3)	16 (11.5)	0.007
EuroSCORE II, %	2 (1)	3 (2)	<0.001
History of AF, *n* (%)	122 (93.1)	133 (95.7)	0.516
NYHA, *n* (%) I, II III, IV	38 (29.0) 93 (71.0)	0 139 (100)	Not applicable
Body mass index, kg/m^2^	27.44 (4.30)	29.00 (7.48)	0.040
H_2_FPEF	6.34 (1.14)	6.79 (1.27)	0.003
LVEDd, mm	49.80 (6.44)	48.48 (5.95)	0.093
LVEF, %	59.05 (5.55)	55.94 (4.13)	<0.001
LA diameter, mm	47.16 (7.10)	44.10 (5.96)	<0.001
AFMR ≤2/4 3/4, 4/4	39 (30%) 92 (70%)	133 (96%) 6 (4%)	<0.001
Tricuspid regurgitation	2.14 (1.03)	1.68 (0.94)	<0.001
Systolic PAP, mmHg	42.04 (10.52)	44.22 (13.62)	0.150

Abbreviations: AF: atrial fibrillation; AFMR: atrial functional mitral regurgitation; COPD: chronic obstructive pulmonary disease; LA: left atrial; LVEDd: left ventricular end-diastolic diameter; LVEF: left ventricular ejection fraction; MDRD: The Modification on Diet in Renal Disease; MVA: mitral valve annuloplasty; NYHA: New York Heart Association; PAP: pulmonary artery pressure.

**Table 2:** Predictors of all-cause mortality, HFpEF readmissions, and their composite.

	Multivariable Analysis	
	HR (95% CI)	*p*-Value
All-cause mortality		
Age	1.05 (1.01–1.10)	0.031
Body mass index	0.93 (0.87–0.99)	0.023
MV repair	0.16 (0.07–0.34)	<0.001
HFpEF readmissions		
Diabetes mellitus	5.14 (1.16–22.73)	0.031
TR grade	2.14 (1.00–4.58)	0.050
MV repair	0.21 (0.09–0.51)	<0.001
Mortality and HFpEF readmissions		
Age	1.03 (1.00–1.07)	0.050
MV repair	0.22 (0.13–0.41)	<0.001

CI: confidence interval; HR: hazard ratio; HFpEF: heart failure with preserved ejection fraction; TR: tricuspid regurgitation; MV = mitral valve.

## Abbreviations

AF	atrial fibrillation
AFMR	atrial functional mitral regurgitation
BMI	body mass index
COPD	chronic obstructive pulmonary disease
HFpEF	heart failure with preserved ejection fraction
LA	left atrium
LAD	left atrial diameter
LV	left ventricular
LVEDd	left ventricular end-diastolic diameter
LVEF	left ventricular ejection fraction
MR	mitral regurgitation
MV	mitral valve
MVRepair	mitral valve repair
NYHA	New York Heart Association
StanCare	standard of care
PAP	pulmonary artery pressure
TR	tricuspid regurgitation
